# College students still maintain the traditional Chinese concept of love

**DOI:** 10.1016/j.heliyon.2023.e13786

**Published:** 2023-02-22

**Authors:** Juan Sun, Fengqing Li, Shiqi Wang, Zeyu Lu, Chengyi Zhang

**Affiliations:** a Inner Mongolia Honder College of Arts and Sciences, Hohhot, Inner Mongolia, China; b Faculty of Public Health, Inner Mongolia Medical University, Hohhot, Inner Mongolia, China

**Keywords:** Love, Prevalence, Traditional Chinese concept

## Abstract

A cross-sectional census was conducted on all students at the campus of Inner Mongolia Medical University using a self-administered questionnaire. This study aimed to investigate their love from psychological pressure, behavioral characteristics, social cognition, etc. Our results show that all students cultivate feelings and become lifelong partners as their motivation for love. Among the population with various demographic characteristics, the prevalence of love among disadvantaged groups is low; that is, women are lower than men, and those from rural are lower than those from cities. After balancing areas and gender, mental working families have a high prevalence of love. Instead, students whose parents' high expectations pressure their children to have a high love prevalence. This is due to China's specific historical perspective. Our results suggested that although China has been deeply integrated with the world and has more frequent ideological and cultural exchanges, college students still inherit China's traditional outlook on love.

## 1 Introduction

Love is part of a suite of human reproductive strategies, particularly in long-term terms. A developed mammalian drive to pursue and keep preferred mates [1] applies to people of all cultures, races, ethnicities, religions, and sexual orientations [2]. Erik Erikson considers loving a successful resolution to the developmental crises of young adulthood [3]. One view about love is that it is a cultural phenomenon, a learned effect or behavioral response resulting from experiences and culture [4]. In old China, the traditional concept of marriage was stereotyped and deeply constrained by feudal ideology [5]. As early as this century, Wendy reported that adolescent sexual activity is influenced by their views of sex and their perceptions of their mothers' views [6]. In ancient China, love is affected by their views and parents. Their parents decide the marriage object of young men and women, and their parents arrange the wedding. Women hardly ever appear in public. When they reach the marriageable age, their parents are responsible for finding husbands. Meanwhile, parents of marriageable men were also responsible for finding a wife. Generally on the wedding day, the bride covers her face with red silk, called ‘gai tou’, during the wedding ceremony. When the bridegroom and bride enter the bridal chamber after the wedding ceremony, the bridegroom can lift the ‘gai tou’. In some cases, the couples meet each other for the first time at this moment.

Therefore, it is impossible to have them fall in love in Chinese arranged marriages [7]. Communication between young men and women has gradually become common with the development of society and the disappearance of feudal ideology. Private contact with the opposite sex is no longer as disturbing as before. The social environment of shared free love gradually formed and various ways of love appeared. Most young men and women express their feelings toward the opposite sex and fall in love. Meanwhile, they also communicate with their parents and take parents’ opinions as references. Love without parental support was considered unhappiness and unacceptable emotionally by young men and women.

The traditional Chinese love concept has been in the process of continuous development. Whether how love changes, the Chinese love concept always advocates the constant marriage from ancient times to the present. In ancient times, a married couple referred to a marriage where both men and women take a hair from their heads and combine it to show that the hair is concentric, love is eternal, they stick together in life and death, and never separate. A love relationship signifies severe and long-term commitment in Chinese traditional culture. It carries the entrustment of elders and the hope of relatives. Marriage is not only for individuals but also for parents and clans. The long-term existence of such a love culture, which we call “constant marriage culture”, makes Chinese people more rational and cautious in a love relationship. Rational people consider more pragmatic factors in romantic relationships than emotional people [2]. “Hold your hands and grow old with you” is a noble yearning for pure love since ancient times.

The Chinese people's deep and implicit view of love has always been maintained. This is the traditional virtue of Chinese people in love. They are peaceful and measured, and love is very disciplined. This poem itself appears in the form of a men's love song about pursuing women. This is probably related to the man being the driving party in the general marriage relationship. Even in modern times, when a girl likes a guy, she always waits for him to speak first.

Since the reforms and opening up, China's economy has developed rapidly [[8], [9], [10]], and the social environment has significantly changed. Meanwhile, western culture has continued to enter China. China is going on the pathway of detraditionalization with specific Chinese features [11]. This change led the traditional love concept and western love concept to co-exist. The population of various social strata and characteristics show various attitudes towards love. Some young men and women who just got rid of the shackles of old Chinese marriage would go to the extreme of a casual attitude toward love under the influence of western ideologies of ‘sexual freedom’ and ‘sexual liberation’. For the new generation of Chinese, romantic relationships, which are considered preparation for long-term commitments or considered a game or short-term emotional experience, are not a matter of morality or discipline [2]. However, more than a quarter of women in some rural areas believe marriage should completely obey their parents' opinions [12]. After all, decades from the reforms and opening up, compared with the thousands of years of the deep richness of traditional culture is a short duration.

Love is influenced by complex cognitive processes and other factors [1]. The cognitive level of people with different levels of education was varied significantly. For university students, their judgment ability is more than peers with lower educational background, and their love concept will not be easily affected by cultural conflict. University students have a higher level of knowledge, they can think rationally about cultural conflicts and independently choose to accept or reject them. One research has shown that cultural differences in communicating love between the US and East Asian countries of China, Japan, and South Korea [13]. It reported that Chinese students have a lower acculturation level to foreign culture (U.S.) compared with students from other Asian countries [14]. Since Chinese students’ love concept is developed based on rich traditional culture. They only absorb the contents of western culture that fit the current Chinese social environment and are suitable for themselves, rather than blindly accept western culture or inherit all traditional culture. For instance, compared with the social status was more highly valued by the parents, romantic love was more highly valued by young men and women [15]. The intimacy and passion of Americans are generally higher levels than that of Chinese in romantic relationships [16]. We consider it necessary to understand the love attitude of university students with high educational backgrounds. We start the study with the data from Inner Mongolia Medical University. Our study indicates that traditional Chinese culture is still deeply rooted in the subconscious of university students. Research has shown that despite a profound social revolution, a relatively conservative sexual culture still exists in China [17]. Personal development is a priority for our students, and it is also the most valued in love. Students fall in love generally for their lifelong happiness. The traditional gender role affects our students. Most female students are cautious, implicit in love and cover up their original feelings. Economic pressure is an obstacle to the love of rural students. Rural students are more worried about the pressure from the economy. Students from manual labor families also have a similar burden to fall in love. Family plays an irreplaceable role in the love of students. Traditional Chinese parents have high expectations of their children and these unreasonably high expectations are as everyone knows [18]. Students fall in love to pour out pressure on their boy/girlfriend to escape the pressure from the expectation.

## 2 Materials and methods

### 2.1 Method

A cross-sectional census using a self-administered questionnaire was conducted among medical students at Inner Mongolia Medical University [19,20]; they lived on campus at the time. A total of 6677 students were enrolled at the Inner Mongolia Medical College campus. Since some students ask for leave or go out, 6044 students for whom complete questionnaires was available (survey rate = 90.52%) at the time of the census [21]. Effective questionnaires were 6037 (effective questionnaire prevalence = 99.3%). The average age was 21 ± 1.5 years. The project was obtained approval from the school's authorities to conduct the study and was approved by the ethical approval committee of Inner Mongolia Medical University (IMMU) and respondents' consent for using a questionnaire to collect data. Four researchers contacted the school counselor and explained the purpose and methods. According to the number of students in each department, one to three academic departments were covered daily. One of the four researchers explained the study's aims to the subjects, assured them that the results would be used for scientific purposes only, distributed the questionnaire, and asked them to complete it anonymously, without looking at their neighbors' answers. Finished questionnaires were placed in a sealed envelope [19].

### 2.2 Questionnaire

This questionnaire is based on 36-Item Short Form Survey Instrument (SF-36). Research Institute Of Health Research In Boston. 1988. Index of Learning Styles Questionnaire (NC STATE UNIVERSITY), from Research Gate. Richard et al. (1999), Patient Health Questionnaire (PHQ-9) (Pfizer, Inc), MSU Olin Student Health Center (1999), and is modified many times according to the specific situation of the school in pre-investigation. The six self-rated items in the questionnaire were tested for reliability and the Cronbach's coefficient alpha was used to evaluate (Alpha = 0.665).

The questionnaire was divided into three parts. The first collected demographic data. Research has shown that the within-person association between romantic involvement and psychological distress varies based on sexual identity and race/ethnicity [22]. Mongolians are the main minority in the Inner Mongolia Autonomous Region in China. The Mongolian ethnicity included Mongolia, Evenki, Oroqin, Hezhen and Daur [23]. Other ethnicities include Menchu, Hui, Korean etc., except Mongolia and Han ethnicity. The ethnic information was obtained from the school database. Faculties are classified into non-medicine faculty and medicine faculty. Medicine faculty includes ‘traditional Chinese Mongolian medicine’ and ‘clinical medicine’ by characteristics of medical education in Inner Mongolia Autonomous Region. ‘Traditional Chinese Mongolian medicine’ faculties include traditional Chinese medicine, traditional Chinese pharmacology, acupuncture and massage, Mongolian medicine, Mongolian pharmacology, Mongolian nursing and ethnic minorities pre-university course. ‘Clinical medicine’ faculties include clinical medicine, forensic medicine, nursing, oral medicine, preventive medicine, pharmacy, clinical pharmacy, anesthesiology, medical imaging, medical laboratory, psychiatry, rehabilitation therapy, applied psychology and pediatrics. ‘Non-medicine’ faculties include public affairs management, biomedical engineering, pharmaceutical marketing, information management and information system, pharmaceutical preparation, and English and marketing students. All of the faculties have four- or five-year programs [23] and most of faculties students have internships that left campus in the last one or two years [20], such as the grade 4 and 5 students rarely stay on campus. The grades are classified into higher and lower grades. The lower grades include grades 1 and 2, and the higher grades include grades 3 and over. We also defined students from a city or suburb as urban, and those from a village or pastoral area as rural [24].

In our study, ‘significant population’ are referred to as the significant difference in multiple logistic regression analysis.

The second inquired about the participants' love lives. Based on our customs, we designed options as follows: (1) I am currently in love. (2) I was in love once before. (3) just in love with someone in the self-heart, but he/she does not know it. (4) I am never in love yet. (5) I do not love anyone during school. (6) I am in a secret relationship. “Secret” refers to the stage when the relationship between the parties is not determined whether they can get married or not, and they are not willing to disclose it to others. In our research, six love items can sometimes be summarized into two or three. “In love” includes ‘currently in love’ (loving), ‘in love once before’ (loved), and ‘a secret relationship’. “Not in love” includes ‘never in love yet’, ‘not in love during school’ and ‘just in love in self heart’.

The third asked about love-related items, including love motives, related psychological factors for love and love related psychological consulting. Inner Mongolia Medical University has a mature psychological counseling center, and services include individual psychological counseling, group psychological counseling, students' mental health files, etc. Psychological consulting be designed as multiple-choice questions, the options include ‘loving’, ‘personal development potential’, ‘academic’ and ‘think psychological consultation is unnecessary’. The answers about motives for love designed seven options and required ranking importance on a scale of 1st to 7th. The “Causes of psychological pressure” item in which their answers are designed concerned about nine options, and required ranking importance on a scale of 1st to 9th.

### 2.3 Statistical analysis

Binary multivariate logistic regression was used to analyze the effect of demographic characteristics to love. This technique is used for analyses with a dependent variable with 2 categories (in this case, love and non-love). Independent variables included gender, grade, faculty, residence, ethnicity, etc. Curd OR value and Adjusted OR value were calculated. Cochran-Mantel-Haenszel test was used to analyze related factors' effect on love after stratified by gender and residence. Test of Homogeneity of Odds Ratio was used to analyze homogeneity of stratification. Mantel-Haenszel chi-square value and the merged OR value are calculated. The consultation prevalence of various items in multiple options of psychological consultation was calculated respectively and a significant difference was detected by Pearson's χ^2^-square test. Furthermore, the love item consultation prevalence by family-related factors and various significant populations was analyzed. Love motivation and psychological pressure were evaluated with an average comprehensive score. The psychological pressure was calculated among the various significant population. The average comprehensive score for ranking questions is: the average comprehensive index = ∑ (frequency × weight)/the number of students who fill in this question. Kendall's rank correlation was used to analyze different levels of love stages students the change psychological pressure.

All the hypothesis testing level of significance has been fixed at 0.05. Data were recorded using EpiData (Epi-Data Association, Denmark; v3.1). Statistical analysis was performed using Microsoft Excel (Microsoft Excel 2019 MSO) and Statistical Package for Social Sciences (SPSS Inc., Χ^2^cago, IL, USA, v25.0).

### 2.4 Intentional options

It is worth emphasizing the importance of family for romantic relationships in young adulthood [25,26]. Adolescents develop interpersonal skills and competencies within the family context, which prepare them for later peer and romantic interactions [24]. Consequently, it is critical to highlight that the practical consequences of studies on romantic relationships in adolescence and well-being must consider the contexts in which adolescents develop [25]. We designed family-related items. Children from different families have a different lot and conduct themselves in society. It has existed from old and is common in various countries. A report pointed out that adolescents perceiving their families to be less well off than others were twice as likely to report sexual abuse as those of ample or medium family affluence. Poverty is a risk factor for sexual abuse [27]. Although it has different forms of expression for different regional environments, living habits, political systems and religious beliefs, unfairness exists everywhere for the children from different families. In our university, students in a class live together and come from different families, which brings differences in students views. The rich children eat and use well, but they are less than those with an ordinary families. This will give students an inferiority complex, giving rise to a series of perplexities; we chose ‘birth family’ items. We designed options such as “Mental labor family’ (including cadre family and intellectual family) and ‘Manual labor family’ (include peasants and herdsmen family, working family, and freelancer family).

In many countries, parents' expectations play an important role in their children's progress [28,29]. China's parents are used to having great expectations for their children. Research from China on educational expectations vs. left-behind children shows that their mental health was worse when parents' educational aspirations were higher than their children's educational expectations [30]. Since they love their children too much, they often compare their children with nearby children and naturally impose their wishes on their children. They want their children to compete for a good place in their studies and progress in their future careers. Indeed, adolescents felt more supported by their friends or parents; they experienced increased happiness and social connectedness [31]. However, the strength and manner of parents' support for their children is a very complex problem [32]. The over wishes lead to many children's rebellious psychology toward their parents and make them feel unhappy and stressed. Therefore, we designed this item. We chose the effects of parents' expectations on students, we designed options as students with “pressure”, “encouragement” and “no matter” from parents' expectations. Since less than 5% of the people answered “no matter”, we ignored it.

Whether or not you can pour out your trouble and to whom you pour out depends not only on your personality but also on your family background, educational background and the environment at that time. “Have to get it out of one's chest” is a kind of release and decompression for your mood and state of mind are much better after talking. Furthermore, if the listener is good enough to give some kind suggestions, the narrator would be completely relieved. Introverted or ‘social phobia’ people are not good at communicating with others and are less likely to reveal their minds. There are chat jobs in some countries. It is considered that some things are easier to say to strangers because they only occasionally appear in their own-self lives, so talkers have no pressure and it is easier to say what they think in the face of them. However, it will be very embarrassing and difficult to accept in China. In order to understand whether talking to others when encountering troubles affect love and the degree related to love, we chose the item ‘willing to pour out pressure’ with options ‘yes’ and ‘no’.

College students are a group of people who are about to enter society. In today's highly developed socialization period, they cannot succeed only by personal skills. They have to face many challenges. If they want to succeed, they should make friends, broaden their horizons, cultivate their good communication skills and enhance their social relations with the people around them. This is the essential requirement for entering an official career, even though during university, making friends is related to the most sensitive issues of today's college students, such as interpersonal relationships, personal future, academic, and adaptation to the environment. Of course, making friends is related to one's personality. Outgoing people who like to chat with others prefer to go to places with many people and who have just met can talk naturally and make friends easily. In this way, we have designed making friends widely with the answer options ‘yes’ and ‘no’.

Being able to pursue the opposite sex actively and talking about sex with others are two items related to loving. For westerners, these behaviors are normal without any concerns, but they were forbidden in ancient China. Even for modern college students, these behaviors still have significant concerns and different degrees of recognition. In ancient times, discussing sex-related topics would be despised and feared due to the limitation of feudal superstition. Over the years, Chinese people's concept of sex has changed. However, due to the lack of sex education, people know little about sex knowledge, so there is little sex talk. During the “Cultural Revolution”, sex became increasingly politicized and almost formed an “asexual” culture. Since the reform and opening up, we had an inclusive attitude towards sex, and the openness of sexual topics has reached a certain degree. However, even now, we can see that when talking involves sexual issues, some people will feel overwhelmed, make a fuss, and even doubt the other side. In this way, we have two questions ‘pursue the opposite sex’ with the answer options are ‘active’ and ‘inactive’ and ‘whether talks about sex with others’ with the answer options are ‘yes’ and ‘no’.

The mental health of children in single-parent families has been a worldwide concern for a long time. In recent years, Japan has reported on the physical, psychological and behavioral problems of children in single-parent families [33,34]. At the beginning of the founding of new China, China officially promulgated the marriage law. At that time, divorce was rare, and single-parent families were also rare. The marriage law in 1980 added “if the emotion has broken down and the mediation does not work, divorce is allowed”. With the relaxation of the national marriage policy, the number of divorces gradually increased, and the children's worries of single-parent families began to appear and become more and more, In China, people are paying attention to it increasingly. Given Chinese historical reasons, college students from single-parent families have their unique characteristics. Many students are unwilling to disclose the fact of their single parents. This is because society discriminates against them to varying degrees, and they think it is a disgrace. Long-term concealment and repression have caused different forms of psychological distortion and the role of different living environments can show a variety of psychological obstacles. They feel inferior and lonely due to the lack of father or mother's love or the misunderstanding caused by living in the stepfather or stepmother's house. On the other hand, due to single parents or being fostered in the family of relatives, especially elders (parents of parents), they think that they are in debt and show excessive doting and lack of necessary education to single-parent children, resulting in their children's selfishness and rebellion. Therefore, we used whether it is a single-parent family in the questionnaire.

China implemented the Family Planning policy from 1980 to 2016, advocating that each family has only one child. The surveyed students at that time were in the historical stage of implementing the family planning policy in China. The students with no siblings have become a huge population during the investigation period. We have listed the item “Students with no siblings”, and the answers are divided into ‘Yes’ or ‘No’.

### 2.5 Results

Among participants, the love students were 60.0%, including 24.0% being in love, 20.7% in loved before, and 15.3% in a secret relationship. In addition, 27.3% of students decided not to fall in love during university.

**Fig. A1:**
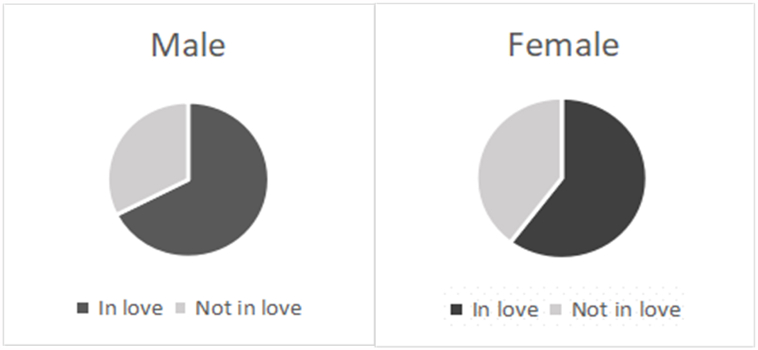
Love prevalence by gender in Inner Mongolia Medical University.

**Fig. A2:**
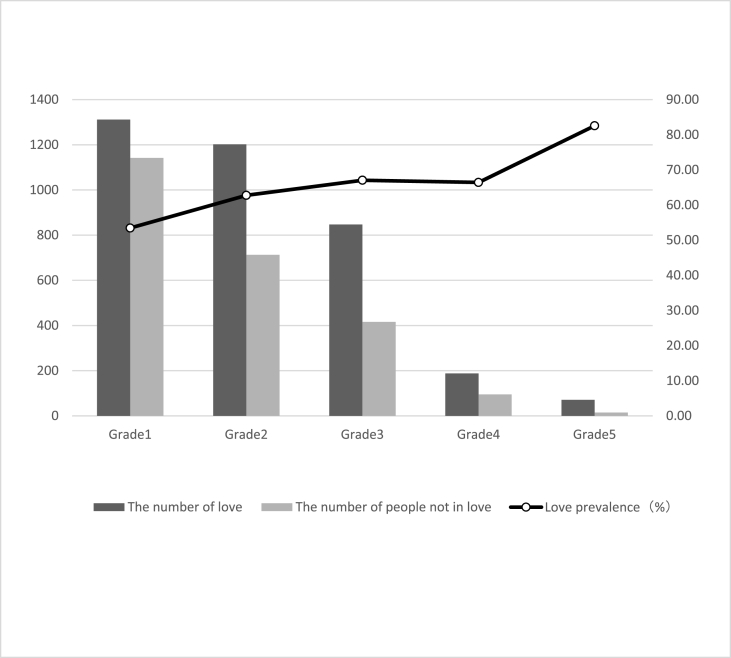
Love of grades in Inner Mongolia Medical University.

**Fig. A3:**
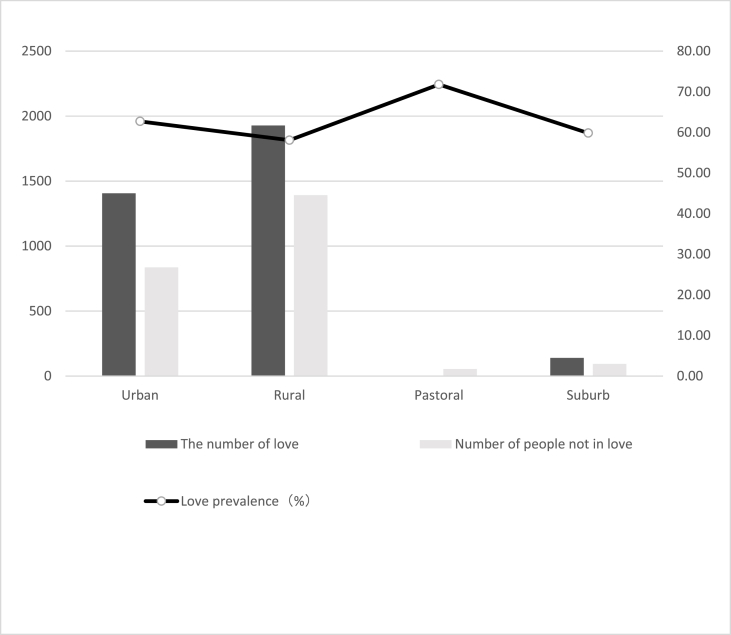
Love number by area in Inner Mongolia Medical University.

**Fig. A4:**
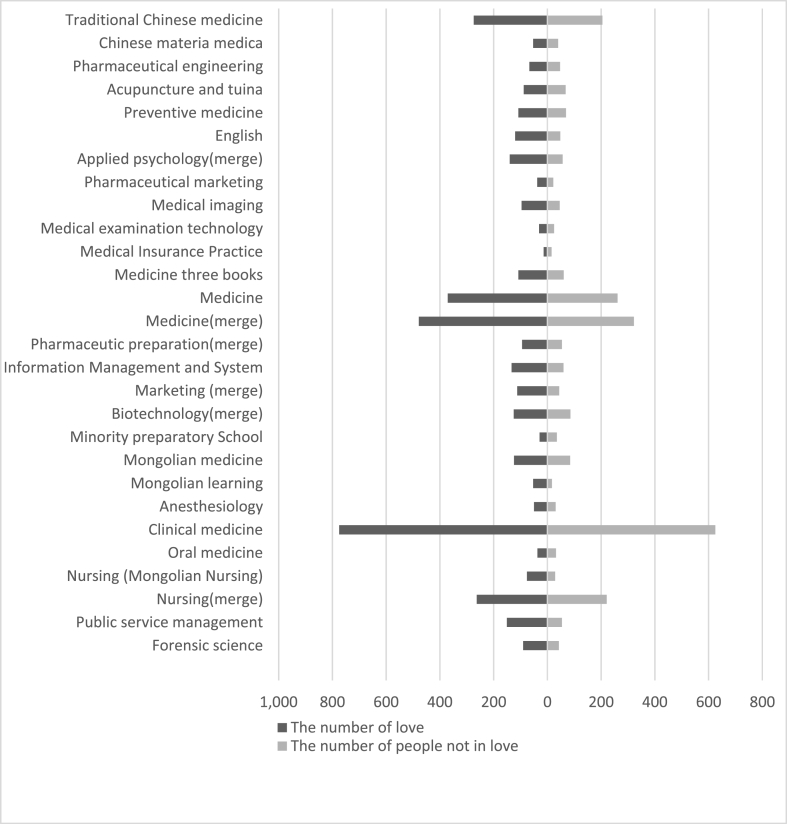
Love prevalence among various faculties in Inner Mongolia Medical University.

**Fig. A5:**
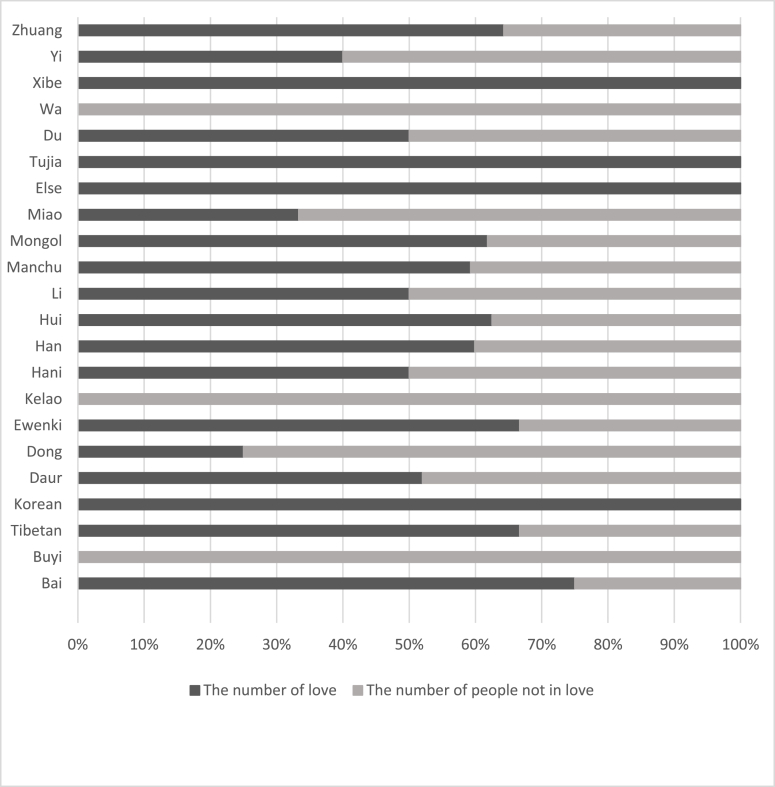
Love prevalence among various ethnic in Inner Mongolia Medical University.

Table 1 shows the love prevalence and logistic regression analysis of demographic characteristics. The love prevalence of male students was 1.14 times that of female students (Fig. A1). Higher grade love prevalence was 17.76% higher than lower grade (Fig. A2). Urban students’ love prevalence was 6.29% higher than rural (Fig. A3). The love prevalence of non-medical faculty is significantly higher than that of medical faculty (Fig. A4). There is no significant difference between different medical faculties (Traditional Chinese Medicine and Mongolian Medicine, Clinical Medicine). Higher grades and non-medicine faculty are the risk factors for love; the risk is 1.48 (95% CI; 1.313–1.676) and 1.44 (95% CI; 1.218–1.711) times of love when compared with counterparts. Women and rural are the protective factors of love, the risk is 0.71 (95% CI; 0.632–0.799) and 0.87 (95% CI; 0.782–0.972) times of love compared with counterparts. There were 23 ethics among the participants. The love percentage of each ethnic is shown in Fig. A5. The love prevalence of Mongolian, Han, and other ethnicities was roughly consistent (59.60%–61.37%). In a word, the love prevalence for students of male, senior, from urban and non-medical is higher.

**Table 1:** Love prevalence and logistic regression analysis by demographic characteristics in Inner Mongolia Medical University.

	n	Prevalence (%)	Crud OR	(95% CI)	Adjusted OR	(95% CI)
Gender						
Male	1165	65.78	1		1	
Female	2455	57.64	0.697	0.621–0.783	0.710	0.632–0.799
**Grade**						
Lower	2514	57.27	1		1	
Higher	1106	67.44	1.551	1.376–1.749	1.484	1.313–1.676
**Faculty**						
Traditional Chinese Mongolian Medicine	697	58.97	1		1	
Clinical Medicine	2148	57.77	0.964	0.844–1.102	1.011	0.876–1.165
Non-medicine	775	68.58	1.444	1.218–1.711	1.436	1.201–1.717
**Residence**						
Urban	1546	62.21	1		1	
Rural	2068	58.53	0.859	0.773–0.955	0.872	0.782–0.972
**Ethnicity**						
Mongolian ethnicity	880	61.37	1		1	
Han ethnicity	2567	59.60	1.084	0.958–1.227	1.176	1.031–1.341
Other ethnicity	173	59.86	0.981	0.778–1.237	0.929	0.734–1.175

Table 2 shows the Cochran-Mantel-Haenszel test of love-related factors by gender. The prevalence of love for women from manual labor families (56.54%) is lower than that of students from mental labor families (65.05%) (χ^2^ = 15.40; P = 0). It affects female students strongly (OR = 1.43). After balancing gender factors, it is still found that different family backgrounds can cause differences in love (χM-H^2^ = 18.999; P < 0.001). Students from mental work families are more likely to fall in love. Among all participants, the number of manual labor families (4540) is five times mental families (904) are. As students from manual labor families dominate, it is inferred that it is one of the reasons for the low prevalence of love in our university. For parental expectations for their children, male students feel that parents' expectations as encouragement (love prevalence 66.07%) and pressure (66.96%) (χ^2^ = 0.12; P = 0.73) do not affect love. The love prevalence of female students who felt pressure (62.10%) was higher than those who felt encouragement (56.84%, OR = 1.24) (χ^2^ = 7.50; P = 0.01). Parental expectation, regardless of gender, affects students' love (OR = 1.175). The love prevalence for students as encouraged only is (59.38%), since they have a stronger desire to study hard, they fall in love relatively less. While the students as pressure fell in love more (the love prevalence 63.84%) (χ^2^ = 8.37; P = 0) to release their pressure.

**Table 2:** The Cochran-Mantel-Haenszel test of love related factors among love students stratified by gender.

	Total	Male			Female			Test of Homogeneity of Odds Ratio	χM-H^2^	P	OR_M-H_	95% CI
	N	n	OR	95% CI	n	OR	95% CI					
**Family**			1.289	0.972–1.710		1.430	1.196–1.711	>0.05	18.999	<0.001	1.388	1.193–1.615
Manual labor	4540	830			1828							
Mental labor	904	195			402							
**Parents' expectations**			1.041	0.830–1.305		1.244	1.064–1.455	>0.05	8.367	0.004	1.175	1.033–1.336
Encouragement	4740	855			1941							
Pressure	1283	306			508							
**Pour out pressure**			0.83	0.667–1.033		0.892	0.776–1.024	>0.05	0.292	0.025	0.873	0.777–0.982
No	1650	364			673							
Yes	4347	800			1780							
**Make friends widely**			0.858	0.665–1.106		0.984	0.834–1.160	>0.05	0.459	0.498	0.945	0.823–1.085
No	5013	961			2050							
Yes	1012	200			399							
**Pursue the opposite sex**			2.151	1.759–2.632		1.874	1.611–2.180	>0.05	152.676	<0.001	1.966	1.742–2.219
Incative	4043	442			1763							
Active	1987	721			688							
**Talk about sex with others**			0.615	0.477–0.792		0.844	0.742–0.961	<0.05	24.011	<0.001	0.792	0.706–0.889
No	4341	994			1690							
Yes	1697	171			764							
**Students from single parent homes**			0.942	0.593–1.496		1.111	0.841–1.4677	>0.05	1.064	0.650	1.064	0.838–1.350
No	5730	1109			2322							
Yes	304	54			132							
**Students with no siblings**			1.006	0.824–1.227		1.119	0.979–1.278	>0.05	5.090	0.024	1.083	0.969–1.209
No	3942	637			1686							
Yes	2092	526			768							

For ‘pour out pressure, we found that different ‘pour out pressure or not’ could cause the difference in love (χM-H^2^ = 4.657; P = 0.031) after balancing the gender factors. The students willing to ‘pour out pressure’ have a lower love prevalence of 59.35% than 62.85% (χ^2^ = 4.66; P = 0.03) for those unwilling to ‘pour out pressure’.

Students who are encouraged by their parents' expectations are more willing to talk to their parents after being pressured (26%), compared with those whose parents' hopes cause pressure on their children (23%) (χ^2^ = 4.43; P = 0.04). ‘Stressed students’ are more willing to talk to their relatives and friends and even other various people except for their parents. More than half (54%) of the ‘stressed students’ confided their pressure on other outsiders, while only 45% in ‘encouraged students’. Since ‘stressed students’ are under pressure at home, it is easier for them to communicate with more people outside, and their opportunities to talk about the pressure are greatly increased, which makes Percentage of ‘keep it in the heart’ rather than ‘pour out pressure’ for ‘stressed students’ is lower than those “encouraging students” who mainly talk about the pressure to their families (χ^2^ = 15.982; P < 0.001). (Table A1).

For ‘the pursuit of the opposite sex’, the love prevalence of the answer positive in men is 73.95%, and the love prevalence of women is 69.08% (χ^2^ = 5.74; P = 0.02), while the answer is negative, 56.89 for men and 54.38 for women (χ^2^ = 0.3; P = 0.58). The average love prevalence of ‘active pursuit of the opposite sex’ (71.49%) was higher than ‘passive pursuit of the opposite sex’ (54.86%) (χ^2^ = 152.68; P = 0). After balancing gender factors, “pursuing the opposite sex” may lead to a difference in love prevalence (χM-H^2^ = 152.676; P < 0.001). Whether gender factors are balanced or not, different numbers of parents do not lead to differences in love. The love prevalence of men in single-parent families is (65.06%) and two-parent families (66.41%) (χ^2^ = 0.06; P = 0.8), women in single-parent families (60.27%) and two-parent families (57.73%) (χ^2^ = 0.55; P = 0.46). The average love prevalence of single-parent families (61.59%) and two-parent families (60.28%) (χ^2^ = 0.21; P = 0.65). For the siblings” in love, the love prevalence of students with siblings is 66.29% for men, 57.04% for women (χ^2^ = 9.23; P = 0) and students without siblings (men 66.41%, women 59.77%) (χ^2^ = 25.70; P = 0). For students with or without siblings, the love prevalence of men is higher than whose of females. The average love prevalence of students with siblings (59.31%) and students without siblings (62.30%) (χ^2^ = 5.09; P = 0.24). Only one child in a family did not affect love after balancing gender factors. However, it is known to be a concern in our society.

Factors contributing to the high love prevalence include “mental labor family” in females; “parents' expectations bring pressure” in females; “pursue the opposite sex”; “not talk about sex with others”. Factors with high love prevalence after balancing gender factors include “mental labor family”; “parents' expectations bring pressure”, “pour out pressure”; “pursue the opposite sex actively”, and “do not like to talk about sex with others”. Whether gender balance or not does not affect the love prevalence, including “single-parent families”; “siblings”.

Table 3 shows the Cochran-Mantel-Haenszel test of love-related factors among love students stratified by residence. Whether students from rural areas or cities, the love prevalence of families with manual labor (58.31% in urban and 59.49% in rural) is lower than families with mental labor (69.17% in urban and 64.80% in rural) (χ^2^ = 15.27; P = 0) (χ^2^ = 5.10; P = 0.02) respectively. After balancing the regional factors, we found that different families could cause the difference in love (χM-H^2^ = 18.038; P < 0.001). Students from mental working families (love prevalence 66.70%) are more likely to fall in love than manual working families (love prevalence 58.9%) (χ^2^ = 18.04; P = 0). As far as parents' expectations of their children, feeling both encouragement and pressure from rural areas have no impact on love (OR = 1.16; 95% CI 0.98–1.38). Students from cities feel pressure on their parents' expectations in love are significantly greater than those who feel encouraged (OR = 1.27; 95% CI 1.046–1.542). Parents in urban areas tend to be more concerned about their children than in rural areas, and students from urban areas are more sensitive. As mentioned above, the love prevalence of students feeling pressure (64.34% in urban areas and 63.51% in rural areas) is greater than students feeling encouragement (58.68% in urban and 59.98% in rural). (χ^2^ = 5.86; P = 0.02) (χ^2^ = 2.91; P = 0.09) respectively. After balancing the urban and rural factors, we found that simple parental expectations impacted the students' love (χM-H^2^ = 8.174; P = 0.004). Compared with the students who felt encouragement (love prevalence 59.46%), the students ‘feeling pressure’ (love prevalence 63.88%) (χ^2^ = 8.17; P = 0) fell in love more to release the pressure, which had nothing to do with coming from urban or rural areas.

**Table 3:** The Cochran-Mantel-Haenszel test of love related factors among love students stratified by residence.

	Urban			Rural			Test of Homogeneity of Odds Ratio	χM-H^2^	P	OR_M-H_	95% CI
	n	OR	95% CI	n	OR	95% CI					
**Family**		1.604	1.264–2.036		1.253	1.030–1.525	>0.05	18.038	<0.001	1.387	1.192–1.614
Manual labor	1074			1583							
Mental labor	258			335							
**Parents' expectations**		1.270	1.046–1.542		1.161	0.978–1.379	>0.05	8.174	0.004	1.208	1.062–1.374
Encouragement	1109			1686							
Pressure	368			442							
**Pour out pressure**		0.959	0.8–1.149		0.803	0.689–0.936	>0.05	5.811	0.016	0.865	0.769–0.972
No	442			592							
Yes	1102			1474							
**Make friends widely**		0.987	0.798–1.221		0.923	0.770–1.107	>0.05	0.548	0.459	0.950	0.827–1.090
No	1223			1785							
Yes	255			342							
**Pursue the opposite sex**		1.973	1.649–2.362		2.129	1.829–2.479	>0.05	152.201	<0.001	2.063	1.837–2.317
incative	909			1294							
active	570			836							
**Talk about sex with others**		0.836	0.698–1.000		0.703	0.606–0.815	>0.05	23.455	<0.001	0.754	0.673–0.845
No	1097			1581							
Yes	384			552							
**Students from single parent homes**		0.835	0.584–1.194		1.176	0.925–1.759	>0.05	0.227	0.634	1.060	0.836–1.344
No	1408			2017							
Yes	72			115							
**Students with no siblings**		1.141	0.964–1.351		1.129	0.978–1.303	>0.05	5.020	0.025	1.134	1.016–1.265
No	935			1388							
Yes	545			744							

For pour-out pressure, the students from urban with willing to ‘pour out pressure’ have a lower love prevalence (62.15%) than that (63.14%) (χ^2^ = 5.51; P = 0.02) for the students with unwilling to ‘pour out pressure’. The students willing to ‘pour out pressure’ have a lower love prevalence of 59.35% than 62.85% (χ^2^ = 5.12; P = 0.02) for those unwilling to ‘pour out pressure’. After balancing the residence factors, we found that different ‘pour out pressure or not’ could cause the difference of love (χM-H^2^ = 5.119; P = 0.024). The love prevalence of ‘active pursuing the opposite sex’ (70.54% in urban, 72. 26% in rural is higher than ‘passive pursuing the opposite sex’ (54.83% in urban and 55.02% in rural) (χ^2^ = 55.92; P = 0) (χ^2^ = 96.61; P = 0) respectively. The average love prevalence of students for ‘active pursuing the opposite sex’ (71.55%) was higher than ‘passive pursuing the opposite sex’ (54.94%) (χ^2^ = 152; P = 0) after balancing the residence factors (χM-H^2^ = 152.201; P < 0.001). Like the birth family results, whether in urban or rural areas or balanced urban and rural factors, the students who ‘actively pursue the opposite sex’ fall in love more.

The love prevalence in urban is similar for single-parent families (55.81%) and two-parent families (60.20%) (χ^2^ = 0.98; P = 0.32), and the love prevalence in rural single-parent families (66.09%) and two-parent families (60.44%) (χ^2^ = 2.21; P = 0.14). After balancing the residence, found no significant difference for single parents or not for ‘love’ (χM-H^2^ = 0.227; P = 0.634).

The love prevalence of students with siblings (urban 58.84%, rural 59.75%) is similar to students without siblings (urban 62.00%, rural 62.63%) (χ^2^ = 2.35; P = 0.13) (χ^2^ = 2.73; P = 0.1) respectively. The students without siblings (the love prevalence 62.36%) are similar to those students with siblings (the love prevalence 59.38%) (χ^2^ = 0.66; P = 0.41) to fall in love. However, after balancing residence, the students without siblings fall in love more (χM-H^2^ = 5.02; P = 0.025).

In short, whether balanced or not, the factors causing higher love prevalence included “from mental labor family”; “pursue the opposite sex”, and “not to talk about sex with others”. Factors contributing to the high prevalence of love, after balanced or in urban, included “parents' expectations bring pressure”; “willingness to pour out pressure”. After balancing residence, the students without siblings fall in love more.

Table 4 lists the motivations of Inner Mongolia Medical University students to love. The top two ranks were consistently observed for ‘Choosing a life partner’ (mean ± SD 3.84 ± 2.38) and ‘Deep feeling’ (3.76 ± 2.37). In each comparison group, the mean range in the top two rank options is from 3.71 to 3.93. They are making preparations for their lifelong life with the attitude of solving life-long events and attach great importance to coordinating the feelings of both sides to the extreme. The rank three for love motivation ‘adjusting to stress from study’ means transferring some academic pressure which is the primary task of all things through love. It carries its future, parents' expectations and public social opinion. Those for the range of the other four options were 2.62–3.59. It is nothing the matter of special worth.

**Table 4:** Motives of loving by significant population at Inner Mongolia Medical University.

	Total			Medicine		Non-medicine		Male		Female		Urban		Rural		Lower grades		Higher grades	
	Rank	Mean	SD	Mean	SD	Mean	SD	Mean	SD	Mean	SD	Mean	SD	Mean	SD	Mean	SD	Mean	SD
Meet psychological or physiological needs	12,279	3.39	2.79	3.34	2.82	3.59	2.68	3.54	2.74	3.32	2.82	3.39	2.79	3.39	2.79	3.30	2.77	3.59	2.83
pass the time of boredom	12,250	3.38	2.80	3.34	2.82	3.53	2.69	3.36	2.82	3.39	2.79	3.35	2.78	3.40	2.80	3.37	2.76	3.39	2.88
Adjusting to stress from study	13,044	3.60	2.53	3.58	2.55	3.68	2.49	3.68	2.53	3.56	2.54	3.54	2.57	3.65	2.50	3.60	2.49	3.60	2.64
Choose a life partner	13,881	3.84	2.38	3.87	2.39	3.74	2.34	3.88	2.37	3.82	2.38	3.93	2.38	3.77	2.37	3.81	2.36	3.91	2.41
Go with the flow	9984	2.76	3.00	2.66	2.99	3.14	2.99	2.92	2.99	2.68	3.00	2.66	2.96	2.84	3.02	2.82	3.00	2.62	2.98
Deep feeling	13,601	3.76	2.37	3.72	2.40	3.92	2.26	3.78	2.36	3.75	2.38	3.81	2.38	3.72	2.36	3.71	2.36	3.87	2.38
Changing destiny	10,610	2.93	2.94	2.84	2.94	3.25	2.96	2.95	2.94	2.92	2.94	2.89	2.97	2.97	2.93	2.98	2.93	2.82	2.98

Table 5 shows the psychological counseling of students at Inner Mongolia Medical University. ‘Students consulted for the personal development potential’ was the highest (63.2%). In today's highly competitive Chinese society, people try their best to develop their personal potential by all means. ‘Academic’, which follows the top one (42.2%) is the basis for creating their potential. For these two items, the counseling prevalence of students who are not in love is higher than that of students in love. (χ^2^ = 4.526 P < 0.05; χ^2^ = 3.985 P < 0.05) respectively. ‘Love’ consumed some of their vigors. The prevalence of counseling in loving students for love items was 1.5 times higher than that students not in loving (χ^2^ = 34.093; P = 0), which showed the greatest difference although consulted for love was the lowest (13.5%) among all kinds of items in all of the students.

**Table 5:** The Prevalence of Psychological consultation among students at Inner Mongolia Medical University.

	Total		Not in love		In love		χ²	p
	n	Prevalence (%)	n	Prevalence (%)	n	Prevalence (%)		
Personal development potential	3808	63.20	1560	64.80	2248	62.10	4.526	0.033
Loving	814	13.50	249	10.40	565	15.60	34.093	0.000
Learning	2542	42.20	1052	43.70	1490	41.10	3.985	0.046
Unnecessary	1497	24.80	575	23.90	922	25.50	1.972	0.160

Table 6 presents the love counseling prevalence as significantly higher among the students who feel the pressure from parents' expectations (15.16%), than that of the students who feel the encouragement from parents' expectations (12.83%) (χ^2^ = 4.609; P = 0.032). The higher love counseling prevalence for stressed students seemed the most obvious since they have a higher love prevalence. The consultation prevalence of ‘personal potential development’ and ‘academic’ from families encouraging students is higher than those with stressed students. Visibly, parents' excessive hope for their children can only make them lose more confidence in themselves. Furthermore, they think there is no need to do psychological counseling more than encourage family students. This means that the pressure of parents has also had a negative effect on students' psychology.

**Table 6:** Psychological consultation prevalence by family-related items in Inner Mongolia Medical University.

	Personal development potential				Loving				Learning				Unnecessary			
	n	Prevalence (%)	χ^2^	P	n	Prevalence (%)	χ^2^	P	n	Prevalence (%)	χ^2^	P	n	Prevalence (%)	χ^2^	P
**Parents' expectations**			62.611	<0.001			5.552	0.062			21.867	<0.001			59.203	<0.001
Pressure	746	58.28			194	15.16			530	41.41			341	26.64		
Encouragement	2867	65.94			558	12.83			1882	43.28			997	22.93		
**Family**			3.157	0.206			2.057	0.358			34.460	<0.001			10.059	0.007
Mental labor	552	61.13			128	14.17			300	33.22			260	28.79		
Manual labor	2901	64.03			602	13.29			1983	43.77			1089	24.03		
**Students from single parent homes**			0.037	0.847			0.033	0.856			0.732	0.392			0.707	0.401
No	3612	63.15			772	13.50			2406	42.06			1425	24.91		
Yes	193	63.70			42	13.86			135	44.55			69	22.77		
**Students with no siblings**			0.139	0.709			1.325	0.250			11.450	0.001			0.046	0.830
No	2478	63.01			517	13.15			1721	43.76			979	24.89		
Yes	1327	63.49			297	14.21			820	39.23			515	24.64		

The counseling prevalence of students from manual labor families is not statistically significant compared to students from mental labor families in the loving item (P > 0.05) (χ^2^ = 0.511; P = 0.475), although the love prevalence of students from mental work families was significantly higher than that from physical work families. Unexpectedly, students from mental manual labor families think psychological consultation is more unnecessary than students from manual labor families. Maybe, students from manual working families have more troubles than those from mental working families, while the ways to solve them are less than those from mental working families, this leads to students from manual labor families believing that it is necessary to do psychological counseling. Consistent with the prevalence of love, we did not find the difference in love psychological consultation prevalence in a family between single-parent and two-parent (χ^2^ = 0.033; P = 0.856), although the superiority position of two-parent families. The children's respondents in two-parent families have a higher than average educational level, are younger, and have lower levels of depression [35]. However, another research suggests that children in two-parent households do not always have lower ratings of behavior problems compared to single-father and single-mother households [36]. A single parent is always a controversial topic. Various documents have been reported. The students with no siblings were concerned about are not different from that families with many children in all items of psychological counseling.

We further analyzed the impact of love counseling prevalence in a significant population (Table A2). For gender, our results showed that the love counseling prevalence of men is much higher than that of women. A study may explain this result from a physiological view. Scanned 16 women and 16 men intensely in love using functional MRI, suggesting that men and women differ in romantic processing information and that it may be more effortful for men to perceive and evaluate romance degree [29,37]. While females have a stronger inclination toward friendships and social goals than males [38]. Women have significantly higher odds of more frequently emailing faculty, discussing career plans with faculty, and more frequently discussing grades and assignments [39]. The most significant difference is the grade item. The love counseling prevalence for higher grades reached 17% (χ^2^ = 24.626; P < 0.001). It is natural that the older they age, the more people fall in love and consult for love.

In short, the necessity of psychological counseling varies greatly in different families and children's reactions to parents' expectations. Parents' expectations pressure students and students born in mental working families to think that psychological counseling is necessary more. The ‘pressure’ students' to love counseling is higher than those of the ‘encouragement’ students, although there is no significant difference, it is close to the significant level. Academic counseling of students from mental work family and ‘pressure’ students is lower, and the ‘pressure’ students' consultation on “personal development potential” is also lower.

Table A3 presents the psychological pressure options by family-related factors and significant population.

In addition to “academic”, students are most concerned about the options directly related to their career, such as “interpersonal relationship”, “personal future” and “unable to adapt to the environment".

Among all the options ‘academic’ is always ranked the top one for our students, and they have achieved promising results. Their achievements are recognized worldwide at the beginning of this century [40]. In addition to ‘academic’, “interpersonal”, “personal future” and “inability to adapt to the environment” which are directly related to their career life are the most concerned options among various students' groups. Moreover, students of different genders, birth families and places of residence, despite the different dimensions of cultural capital, have no difference in their cognitive level, and their psychological stress index is similar. The research results from another perspective of Petra et al. [41] did not show any substantive differences in the intermediary mechanism between men and women, low-status and high-status students. As for family and economy, the index ranked next. Until now, China still has parents to provide financial assistance to university children, so the students have not felt particularly serious about economics and are grateful to their families. For love-related options, the students' psychological pressure is weak because many students do not fall in love with the university. They have no pressure on love-related, which is affected by the traditional consciousness of ‘starting a career before starting a family’.

For gender, the psychological pressure gap for ‘rejected by the opposite sex’ between women and men (ratio = 1.48), although men and women consistently put career first and love related last. Once the other party rejects men, they have to bear much greater psychological pressure than women. Consistently, men's psychological pressure on “love” is greater than women's. After all being rejected by the opposite sex is painful. When partners more routinely influence the academic success or other outcomes (e.g., drinking, drug use), peer relationships begin to recede in importance [42]. Men invest more, the harm will be greater. Women's economic stress index is higher than men's, since the primary source of income comes from their parents during college, and women are more likely to worry about their parents than men.

For residents, rural areas' economic and family pressure index is higher than urban areas. The ratio of economic pressure index ranked first in all options (0.89). Life in urban areas is much better than that in rural. This stress gap is also reflected in love-related options. Furthermore, rural students also show greater psychological pressure than urban students to adapt to the new environment and get along with the people around them.

The apparent psychological pressure difference for faculty is ‘rejected by the opposite sex’ (ratio = 0.84) between non-medicine and medicine faculty. This may be related to the large class hours and the heavy learning burden of medical students. For grades, the psychological pressure of higher grades in ‘Interpersonal’ and ‘academic’ is greater than that of lower grades, resulting from accumulated life experience. The two love-related options that cause students between pressure and encouragement for parents' expectations show the greatest difference. Even if the stressed students fall in love, they also have more pressure to be encouraged. They fall in love not so much to reduce the pressure caused by their parents' expectations but rather as a transfer of pressure. For families, the economic pressure and family-related pressure of students from manual labor families are much higher than those of mental labor families. The general income of manual labor families is relatively low, consistent with the pressure caused by urban and rural production. There is little difference for students from single-parent families and no siblings of psychological pressure.

Items related to career include “Academic” “Interpersonal” “Unable to adapt to the surrounding environment” and “Personal future” ranked first. “Economic” “Family” ranked next. “Love” and “Rejected by the opposite sex” ranked last. The economic pressure index in students from rural areas and manual labor families increased significantly. The ‘gender’ and ‘faulty’ difference is the biggest in “Rejected by the opposite sex”. ‘Parents expectations have the greatest difference in love-related items.

Table 7 presents the psychological pressure index of students among various love stages. For psychological pressure order, academic (Average = (In love + Loved + Not in love)/3 ± SE; 5.42 ± 0.04), interpersonal (5.29 ± 0.07), personal future (5.08 ± 0.06), and unable to adapt to the environment (5.11 ± 0.12), t these four items are among the top four, it can be seen regardless of whether they are in love or not, students take career life as the first, and then consider love. Notably, ‘love’ ranked the fifth item among the students who are in ‘currently in love’, ‘in love once before’, while the students with ‘not in love’ are the penultimate item. Students with love experience are more stressed about love-related problems. Students who have been in love once had the pain of being ‘rejected by the opposite sex’, so they think this is serious. For love stages order, the psychological pressure on ‘Academic pressure’, ‘Personal future’ and ‘The future of the country’ decrease with the students love stages: from ‘not in love’ to ‘loved’, and ‘in love’. Students in love pay less attention to surrounding matters.

**Table 7:** Psychological pressure index of students among various love stages in Inner Mongolia Medical University.

	Loving	Loved	Not in love	P	Range
Academic	5.39	5.40	5.47	<0.001	−0.08
Interpersonal	5.22	5.35	5.32	0.602	−0.10
Personal future	5.03	5.08	5.14	<0.001	−0.11
Unable to adapt to the surrounding environment	4.98	5.21	5.14	0.602	−0.16
Love	4.98	5.06	4.07	0.602	0.91
Economic	4.92	4.96	4.82	0.602	0.10
Family	4.75	4.94	4.89	0.602	−0.14
The future of the country	4.04	4.09	4.20	<0.001	−0.16
Rejected by the opposite sex	2.75	3.26	2.51	0.602	0.24

## 3 Discussion

The love prevalence in our universities is similar to that of several universities in Hainan Province in China (58.66%) [43]. It is lower than that in European countries and regions teenagers (81.2%), although they are younger [44], and also lower than that in India (66.7%) [45]. Our students have less love experience than foreign countries considered to fall in love early [44]. It is unacceptable to fall in love before adulthood in China, and family, society and school oppose it. University students, have more attention to academic and potential personal development since young than the general population, so they are relatively old when they start trying to fall in love. It is just like a Chinese proverb called ‘First thrive and then wive’. The most psychological consultation for ‘personal development potential’ and the greatest psychological pressure for ‘academic’, ‘interpersonal’ etc. Are related to life and career. Not only academic but also interpersonal relationships are important. It is well known that the relationship with peers is negative. If there are no friends, lack of interaction with friends, and are not accepted by friends, it will also lead to adolescent depression [46].

Only a very few students consult about love-related items. The traditional Chinese psychology of falling in love means they would rather put love in their hearts than talk with others. In addition, students who fall in love consult more than students who never fall in love. It is natural for students in love to pay more attention to love; on the other hand, it suggests that they attach great importance to it. Marriage is also called a ‘life-long event’ in China. Students who fall in love do love psychological counseling to find a better way to maintain their long-term love relationship. This concept is reflected in the greater psychological pressure of love among students with love experience in our study, after all, love is a prerequisite for marriage. Chinese advocate and practice a “constant marriage culture”, which means loving only one person and marry with one person in one's life. This sense is strongly given expression to in love motivation in our study. The top two motivations of love are ‘Choosing a life partner’ and ‘Deep feeling’ in various student populations in our survey. This follows the Chinese love model; once a love relationship is established, the goal is to get married and to live together forever. The two parties in the love relationship often have a great obsession with love in China, which is presented regarding love as the essential affair of a final settlement one's life [47]. Once one loses love, it has lost the power and direction of life [47]. Love is also related to personality. Those who are willing to actively pursue the opposite sex, whether male or female, and students from rural or urban areas, have a higher love prevalence than those who do not seek actively pursuer.

The current national gender equality system has effectively improved women's political status, social status and discourse power [48]; however, gender inequality still exists in the domestic field. The feudal thought of “men being superior to women” has been deeply ingrained and seems to have gradually faded. It still exists in women's subconscious. Women's sense of inferiority is driven by Chinese customs that have lasted for thousands of years. It is a psychological vulnerability caused by the spiritual culture, although the magnitude of this impact varies among populations with different demographic characteristics in our students. Material benefits cannot make up for this psychological barrier for women. The system of equality between men and women cannot fundamentally realize the spiritual equality between men and women. Men's mental health appears to benefit more from the experience of living in a high gender equity society than does women's mental health [49]. Traditionally, men have been considered having multiple partners a positive thing, while women are ashamed of it [50]. Under the long feudal history, women have always been attached to men in love and marriage [47]. Our students follow this idea.

Men are more active than women and usually dominate in love. Men also take more initiative in consulting love than that women. Women are usually more passive and shyer when expressing love than men. One research reported that men are three times more than their partners said ‘I Love You’ firstly [51]. The Chinese style of men-dominant marriage is common in China's social environment. In the United States, the men-dominant in Asian men -White women marriages is less than that in Asian men-Asian women marriages [52]. It can be seen that whether men dominate marriage is restricted by certain conditions. Both husband and wife have the same traditional Chinese cultural background, marriage is more likely to be dominated by men. The advantage of men in the traditional Chinese concept of love also brings about the concept that ‘men are responsible for supporting their family and women is only responsible for “assisting their husbands and educating their children”. Men’ domestic roles make men face greater psychological pressure than women in love-related item. Unlike men's responsibility of supporting the family, taking care of the family is considered the biggest ultimate destination of women, women's value is limited in the family [47]. In the traditional Chinese concept, the main task of women is considered to continue the family, bringing the loss of women's discourse power in marriage and love [47]. Women have more misgiving about love than that men. The gap of position between men and women in traditional concepts makes women more cautious in choosing their love partners, just like the Chinese proverb says, “Women are afraid to marry unsuitable men”. This is one of the reasons why the love prevalence of women in Inner Mongolia Medical University is lower than that of men.

There is a huge gap between urban and rural areas in China [53]. Economic difficulties will have a certain negative impact on romantic relationships [54]. A report shows that college students' dropout is not only related to mental health-related but also related to economic factors [55]. Our study showed that urban students had a higher love prevalence than rural students, who were more concerned about the economic impact. In rural, many multiple-child families can only support one child to study at the expense of other children's study opportunities. Students from rural families who are supported to study by their families faced greater economic pressure than urban students during the study period. Economic pressure is the biggest gap in students between urban and rural. The economic pressure makes rural students have no extra money, making them less likely to fall in love. Traditionally, ‘when a little bird grows up, it feeds food to its mother’ advocated by Chinese, rural students to support their families is natural. Besides, just like that Chinese saying, “Receiving water drips when in need, and I shall return the kindness with a spring”. Students from rural also need to redouble pay back what their family has paid for their study. Once in their spare time, they have to work to help their families make a living. Therefore, Love has become a luxury for rural students due to the great pressure of the economy and family. In addition, students from rural bear more psychological pressure when they try to adapt to the new surrounding environment. Unable to adapt to the surrounding environment may cause by the barriers in crossing the living environment from rural to urban; the barriers increase the social cost. For instance, African and Latin American students used to have a burden of ‘acting white’ when they tried to integrate into the dominant white culture in schools and worried about trying too hard to be unpopular [56]. Our rural university students may have a similar burden when integrating into the urban environment. For love, the increase in social caused cost is reflected in the fact that rural students may worry about “not deserve partner” when they fall in love with urban students. Especially Chinese tradition pays attention to “a marriage between families of equal social rank”. Rural students feel a sense of inferiority because they think they have a low family background. Therefore, rural students easily fall into a love dilemma, even being discriminated against in love. This may be one of the reasons why the prevalence of love among rural students is lower than in urban students. The psychological love pressure of students from rural areas is higher than urban students, but there is no difference in the prevalence of love counseling. Students from rural areas are more reluctant to talk about gender-related things. It can be seen that rural students are more depressed and have to bear a greater psychological burden than urban students. We found that the above influence of residence on love in women is more serious than in men. Rural women who treat love are not only more conservative and passive but also more easily feel a sense of inferiority compared with other relevant populations. The higher education institutions should take targeted interventions on the mental health of college students [57]. Recently research reported that the imbalance and disharmony between urban and rural development is the most prominent structural contradiction in China's economic and social development at this stage, rural Per Capita Disposable Income (PCDI) is far lower than urban PCDI [53]. We speculate that with narrowing the gap between urban and rural, the above phenomenon among rural students may gradually weaken, like research that the burden of ‘acting white’ for African and Latin American students has disappeared [56].

For families, the prevalence of love with parents' pressure is higher than with parents' encouragement. The effect of family on the love of men and women in young adulthood is not just around the love period, this kind of effect is long-term, even from adolescence [25]. Our results showed that students who bear the pressure of parents' expectations would choose to fall in love to relieve the pressure rather than to relieve the pressure through psychological counseling. They believe that psychological counseling is unnecessary. Traditionally, the students most choose to pour out pressure on their parents. However, when students are under pressure expected by their parents, they are more willing to talk about the pressure with boyfriends or girlfriends and even all kinds of people rather than with their parents. In addition, a student who bears the pressure of parents' expectations will be committed to maintaining their love relationship, so ‘love’ and ‘rejected by the opposite sex’ bring them greater psychological pressure. The “constant marriage culture” is deeply rooted, especially in the older generation of parents. Even in the face of unfair treatment in marriage, they will choose to endure [47], and students would still find the same attitude in “love tolerance” as their parents. Chinese parents believe in “expecting their sons and daughters to be successful”. A comparison between Asian Americans (predominantly Chinese American) and white youth pointed to Asian parents' unreasonably high expectations [58]. Moreover, Asian Americans who do not meet their parents' definitions of success may particularly suffer [59]. Parents placed their selves unfulfilled expectations on sons and daughters. Students from manual labor family a lower prevalence of love and a more frequent consultation prevalence of academic items means they put energy into academic rather than love compared with students from mental labor family. The high psychological pressure from the economy and family hinders their love for students from manual labor family. Maintaining a long-term love relationship and being responsible for the partner of love for a lifelong one, needs an economic foundation and family support. However, simple romantic love is praised of sing.

In this paper, we addressed the role of the special family in love. We speculate that ethnic minority families, single-parent families, especially one-child families, may have great abnormal compared with students from ordinary families; however, our results do not support this expectation. The students with no siblings grew up alone from childhood, their interpersonal network is relatively simple. They are lonely or lack friends. However, compared with other students, except those without siblings who seem slightly more likely to fall in love, their love-related items are consistent with those of students from ordinary families. The reason may be that students with no siblings are common in the society in China, it is no longer a special population. It was known as early as the last century, young adults of different ethnicities or races who live in the same region have unique dating patterns [60,61]. Inner Mongolia is a inhabit region of Mongolia ethnicity. We compare the love prevalence of Mongolian ethnicity and Han ethnicity students for various problems arising from different nationalities living in the same region [62]. No significant difference in the love prevalence of Mongolian and Han ethnicity in our study has been found since Mongolian ethnicity and Han ethnicity live together similarly for a long time and gradually integrate into the process of long-term intermarriage.

Love prevalence in medical faculty students is lower than in non-medical faculty students. One of the reasons is that the curriculum arrangement of non-medical faculty is less than that of medical faculty at Inner Mongolia Medical University. Medical curricula are challenging in terms of volume and complexity [63]. Therefore, medical faculty students have less time and energy to fall in love. We found that higher-grade students are more likely to fall in love than lower-grade students. This is a natural physiological phenomenon. There is also a saying that “When a son is grown, he takes a wife, and when a girl is old enough, she goes to her husband”. Higher grade students fall in love more, which has led to the emergence of love-related disturbing and intractable problems. Therefore, higher grade students are more likely to consult love than a lower grade.

## 4 Study implications

Our research contributes to College Students' love of literature from three aspects.

First, our research shows that among various Inner Mongolia Medical University groups. However, the way of expressing love may vary based on the environment and location, no matter what kind of love, showing a unified traditional Chinese love motivation; that is, their attitude towards love reflects a single ideological sense. The traditional culture on love inveterate sustained and steadily is rooted in our students' subconsciousness and still affects our students' love. The Chinese love mode of “implicit expression”, “deep emotion” and " Lifelong companionship” are the characteristics of our students' love relationship. Going through decades of reform and opening up, China has been deeply assimilated into the world and in the process of uninterrupted integration of world culture, the ongoing infiltration of Western ideology and values, college students may act out a variety of love ways, which seems to emulate the western pattern of love. It is still boiled down to an in one's heart of hearts Chinese traditional cultural perception – eternal love.

Given the existence of this subconscious, suffering from the attack of ‘being rejected by the opposite sex’ is much greater than ordinary thinking. For permanent “love”, the love advocated by the Chinese people is not like romance, let alone simple dating, but mutual tolerance, care and understanding of each other. Love was regarded as a kind of dedication or contribution to their faith. For the future of the family, one conceals the suffering in his heart and bears it himself. Therefore, Love was vividly compared to “Various flavors” rather than ‘sweetness’ only. In other words, there are troubles and pain in addition to well-being and happiness.

Secondly, the love prevalence differences between men and women, cities and rural areas, and so on have reflected a series of perplexities for love in disadvantaged groups. These differences and the consequent psychological pressure related to love to embody changes in love counseling. Although falling in love is a normal desire of humans, those students who are more likely to experience inferior positions, such as female students, students from rural areas, and students born into manual labor families have greater psychological pressure, resulting in less possibility of falling in love. However, love is a special role in various disadvantaged groups, for love may be used as a deterrent factor besides an attraction factor. Positive love may be a way to remove the disadvantageous status among these college students by trying to help them straighten out their mentality and hint at ways to extricate themselves from inferiority or dissatisfaction. It can also be a social driving force, which may change their own adverse life events and situations and improve their role in society. Our research helps to understand various kinds of troubles and thorny situations that may be encountered by various disadvantaged groups when they fall in love. All these analyses provide insights into helping clarify and extend their lives track which is more meaningful to policymakers.

Finally, our research also reveals the conflict between material culture and love, a new manifestation of China's rapid economic development and rich material wealth in recent years. In just a few decades after the founding of new China, when the economy and materials were underdeveloped, college students focused more on their feelings for love. They could get married simply without spending much money. Our current research emerges that economic strength is the key factor restricting the progress of love. The economic gap between urban and rural areas reflects this present situation. Due to our data being cross-sectional in nature, these findings cannot be interpreted as causality and need to be considered carefully. Our study can only provide a reference for observing the current situation of College Students' love. Nevertheless, these results suggest that when considering the appropriate specific actions, how college students better adapt to the environment, face reality, and change their mentality may be very useful, which may affect them to achieve their future love goals.

## 5 Limitations

There are several limitations to this study. We missed much important information in the measurement of love, such as the situation of respondents' love: how long has the love lasted before or after entering the university. The number of times they have been in love, whether they break up or are rejected by the other party, and the age at which the students first fall in love. For traditional Chinese students, the harm caused by these problems may be lifelong.

Some potentially important love-related variables still need to be studied. A still promising direction is to consider the intersection between gender widely, race (residence) and social class and so on [40] by⟪latent class analysis⟫ [64], when Inner Mongolia catches up with China's rapid economic development and international exchanges are becoming more and more common. This line of research can better identify the disadvantaged groups in higher education and find the common root causes of various gaps with disadvantaged groups. The research direction [40] put forward a long time ago is still applicable to further research in our university.

## 6 Conclusion

The students' Outlook on love has its own characteristics: “starting a care before starting a family”; “constant marriage culture”; “men being superior to women”; “A marriage between families of equal social rank” etc. College students put academic, personal development and interpersonal the first, indicating the traditional style of ‘starting a career’. Their love motivation is ‘choosing a life partner ‘and ‘deep feeling '. The goal is to get married and “hold your hands and grow old with you”, which shows that the mind retains a “constant marriage culture” and regards marriage and love as a major event in life, “the main affair of their life – marriage”. Students' love expression is implicit and even inhibits their emotional expression, reflecting the traditional Chinese thought of “conservative”. Although the status of women has improved, women have always been attached to men, and men's dominant position in family and love is still deeply rooted. Women are only responsible for “assisting their husbands and educating their children”, and women rarely take the initiative in love, fearing that love for the wrong person will destroy their life; Namely, “women are afraid to marry unsuitable men”. Family background is an important factor in love today. The love prevalence of students from rural families is significantly lower than those students from urban. Under the pressure of economic pressure, students from rural are not easy to fall in love and worry about “not being worthy of a partner”. Love prudence is related to the traditional Chinese “matching families”. Students from rural areas must work to help their families in their spare time. It is also a traditional Chinese concept that “receiving water drips when in need, and I shall return the kindness with a spring”. After all, love needs to be responsible for the lover to the end, and needs the financial foundation and family support. For Chinese parents, “expecting their children to be successful” is eager. They give students too high expectations, resulting in psychological pressure on students. The purpose of parents' marrying their children to their partners is to continue their offspring and be responsible for their ancestors and future generations. This is the traditional marriage thought of the Chinese people. Therefore, today although college students seem to fall in love freely, they still keep the traditional Chinese view of love subconsciously. China has a history of 5000 years. It is deeply influenced by society and family. It is impossible to change these customs in just a few decades. Moreover, according to the rule of the survival of the fittest, the reason why these customs can be maintained to the current day must have its merit, that is, adapted to the environment and cultural characteristics of China.

## Author contribution statement

Juan Sun: Conceived and designed the experiments; Wrote the paper.

Fengqing Li: Performed the experiments; Analyzed and interpreted the data.

Zeyu Lu: Contributed reagents, materials, analysis tools or data; Wrote the paper.

Chengyi Zhang; Shiqi Wang: Contributed reagents, materials, analysis tools or data; Analyzed and interpreted the data.

## Funding statement

This work was supported by 10.13039/501100004763Natural Science Foundation of Inner Mongolia [2016MS0821 & 2013MS1193].

## Data availability statement

Data included in article/supp. material/referenced in article.

## Declaration of interest's statement

The authors declare that they have no known competing financial interests or personal relationships that could have appeared to influence the work reported in this paper.

**Table A1:** The effect of parents' expectation on pour out pressure

	Pressure(n)	%	Encouragement (n)	%	χ^2^	p
Keep it in the heart	297	23.19	1257	28.86	15.982	<0.001
Parents	294	22.95	1126	25.86	4.431	0.035
Relatives and friends	257	20.06	789	18.12	2.478	0.115
Opposite sex	137	10.69	441	10.13	0.348	0.555
Teacher	95	7.42	285	6.54	1.197	0.274
Senior	97	7.57	221	5.07	11.598	0.001
Professionals	104	8.12	236	5.42	12.26	<0.001
**Total**	1281	100	4355	100	50.977	<0.001

**Table A2:** The prevalence of Psychological consultation of love item in Inner Mongolia Medical University

	n	Prevalence (%)	χ^2^	p
**Faculty**			0.026	0.871
Medicine	657	13.47		
Non-medicine	156	13.65		
**Residence**			0.005	0.941
Urban	334	13.46		
Rural	477	13.53		
**Gender**			5.377	0.020
Male	267	15.10		
Female	547	12.86		
**Grade**			24.626	<0.001
Lower	534	12.18		
Higher	280	17.09		
