# Supplementary material for: College students still maintain the traditional Chinese concept of love
Source: Heliyon. 2023 Feb 22;9(3):e13786. doi: 10.1016/j.heliyon.2023.e13786 (PMC10006721; doi:10.1016/j.heliyon.2023.e13786)
Supplement: Multimedia component 1 [file mmc1.docx]

| **TA1: The effect of parents' expectation on pour out pressure** | | | | | | |
| --- | --- | --- | --- | --- | --- | --- |
|  | Pressure(n) | % | Encouragement(n) | % | χ² | p |
| Keep it in the heart | 297 | 23.19 | 1257 | 28.86 | 15.982 | <0.001 |
| Parents | 294 | 22.95 | 1126 | 25.86 | 4.431 | 0.035 |
| Relatives and friends | 257 | 20.06 | 789 | 18.12 | 2.478 | 0.115 |
| Opposite sex | 137 | 10.69 | 441 | 10.13 | 0.348 | 0.555 |
| Teacher | 95 | 7.42 | 285 | 6.54 | 1.197 | 0.274 |
| Senior | 97 | 7.57 | 221 | 5.07 | 11.598 | 0.001 |
| Professionals | 104 | 8.12 | 236 | 5.42 | 12.26 | <0.001 |
| **Total** | 1281 | 100 | 4355 | 100 | 50.977 | <0.001 |

| **TA2: The prevalence of Psychological consultation of love item in Inner Mongolia Medical University** | | | | |
| --- | --- | --- | --- | --- |
|  | n | Prevalence（%） | χ² | p |
| **Faculty** |  |  | 0.026 | 0.871 |
| Medicine | 657 | 13.47 |  |  |
| Non-medicine | 156 | 13.65 |  |  |
| **Residence** |  |  | 0.005 | 0.941 |
| Urban | 334 | 13.46 |  |  |
| Rural | 477 | 13.53 |  |  |
| **Gender** |  |  | 5.377 | 0.020 |
| Male | 267 | 15.10 |  |  |
| Female | 547 | 12.86 |  |  |
| **Grade** |  |  | 24.626 | <0.001 |
| Lower | 534 | 12.18 |  |  |
| Higher | 280 | 17.09 |  |  |

**TA3.: Psychological pressure index among significant population and family in Inner Mongolia Medical University**

| **Psychological pressure** | **Total** | | **Residence** | | | | **Gender** | | | | **Faculty** | | | | **Grade** | | | | **Parents' expectations** | | | | **Family** | | | | **Single parent family** | | | | **No siblings** | | | |
| --- | --- | --- | --- | --- | --- | --- | --- | --- | --- | --- | --- | --- | --- | --- | --- | --- | --- | --- | --- | --- | --- | --- | --- | --- | --- | --- | --- | --- | --- | --- | --- | --- | --- | --- |
|  | Index | Index Rank | Urban | Rural | Ratio | Ratio Rank | Male | Female | Ratio | Ratio Rank | Medicine | Non-medicine | Ratio | Ratio Rank | Lower | Higher | Ratio | Ratio Rank | Encouragement | Pressure | Ratio | Rank | Mental labor family | Manual labor family | Ratio | Rank | No | Yes | Ratio | Rank | No | Yes | Ratio | Rank |
| Academic | 5.42 | 1 | 5.47 | 5.38 | 1.02 | 6 | 5.21 | 5.51 | 0.95 | 6 | 5.42 | 5.41 | 1.00 | 9 | 5.36 | 5.58 | 0.96 | 2 | 5.43 | 5.42 | 1.00 | 9 | 5.46 | 5.41 | 1.01 | 8 | 5.41 | 5.74 | 0.94 | 3 | 5.40 | 5.46 | 0.99 | 8 |
| Interpersonal | 5.28 | 2 | 5.21 | 5.33 | 0.98 | 6 | 5.02 | 5.39 | 0.93 | 3 | 5.30 | 5.23 | 1.01 | 8 | 5.21 | 5.48 | 0.95 | 1 | 5.27 | 5.31 | 1.01 | 8 | 5.17 | 5.33 | 0.97 | 6 | 5.29 | 5.06 | 1.05 | 4 | 5.35 | 5.14 | 1.04 | 4 |
| Unable to adapt to the surrounding environment | 5.09 | 3 | 5.04 | 5.13 | 0.98 | 6 | 4.74 | 5.23 | 0.91 | 2 | 5.03 | 5.30 | 0.95 | 4 | 5.11 | 5.04 | 1.01 | 6 | 5.11 | 5.02 | 0.98 | 6 | 5.35 | 5.02 | 1.07 | 3 | 5.09 | 4.97 | 1.02 | 8 | 5.02 | 5.21 | 0.96 | 4 |
| Personal future | 5.08 | 4 | 5.14 | 5.05 | 1.02 | 6 | 4.98 | 5.12 | 0.97 | 7 | 5.13 | 4.88 | 1.05 | 4 | 5.08 | 5.10 | 1.00 | 8 | 5.08 | 5.18 | 1.02 | 6 | 4.93 | 5.13 | 0.96 | 5 | 5.08 | 5.32 | 0.95 | 4 | 5.14 | 5.00 | 1.03 | 5 |
| Economic | 4.89 | 5 | 4.56 | 5.12 | 0.89 | 1 | 4.75 | 4.94 | 0.96 | 4 | 4.82 | 5.17 | 0.93 | 2 | 4.86 | 4.95 | 0.98 | 5 | 4.83 | 5.12 | 1.06 | 5 | 4.20 | 5.02 | 0.84 | 1 | 4.87 | 5.10 | 0.95 | 4 | 5.04 | 4.59 | 1.10 | 1 |
| Family | 4.84 | 6 | 4.65 | 4.98 | 0.93 | 2 | 4.86 | 4.83 | 1.01 | 8 | 4.83 | 4.92 | 0.98 | 7 | 4.83 | 4.88 | 0.99 | 6 | 4.77 | 5.19 | 1.09 | 3 | 4.34 | 4.99 | 0.87 | 2 | 4.83 | 5.03 | 0.96 | 7 | 4.92 | 4.69 | 1.05 | 2 |
| Love | 4.64 | 7 | 4.54 | 4.71 | 0.96 | 3 | 4.68 | 4.63 | 1.01 | 8 | 4.59 | 4.84 | 0.95 | 4 | 4.60 | 4.76 | 0.97 | 4 | 4.55 | 5.01 | 1.10 | 1 | 4.42 | 4.70 | 0.94 | 4 | 4.64 | 4.65 | 1.00 | 9 | 4.68 | 4.56 | 1.03 | 5 |
| The future of the country | 4.11 | 8 | 4.05 | 4.17 | 0.97 | 5 | 4.28 | 4.04 | 1.06 | 4 | 4.07 | 4.31 | 0.94 | 3 | 4.07 | 4.23 | 0.96 | 2 | 4.19 | 3.91 | 0.93 | 4 | 4.00 | 4.13 | 0.97 | 6 | 4.13 | 3.82 | 1.08 | 2 | 4.18 | 4.00 | 1.05 | 2 |
| Rejected by the opposite sex | 2.76 | 9 | 2.70 | 2.81 | 0.96 | 3 | 3.59 | 2.42 | 1.48 | 1 | 2.66 | 3.18 | 0.84 | 1 | 2.76 | 2.77 | 1.00 | 8 | 2.68 | 2.96 | 1.10 | 1 | 2.75 | 2.76 | 1.00 | 9 | 2.77 | 2.55 | 1.09 | 1 | 2.77 | 2.76 | 1.00 | 9 |
